# Molecular Genetic Influences on Normative and Problematic Alcohol Use in a Population-Based Sample of College Students

**DOI:** 10.3389/fgene.2017.00030

**Published:** 2017-03-15

**Authors:** Bradley T. Webb, Alexis C. Edwards, Aaron R. Wolen, Jessica E. Salvatore, Fazil Aliev, Brien P. Riley, Cuie Sun, Vernell S. Williamson, James N. Kitchens, Kimberly Pedersen, Amy Adkins, Megan E. Cooke, Jeanne E. Savage, Zoe Neale, Seung B. Cho, Danielle M. Dick, Kenneth S. Kendler

**Affiliations:** ^1^Virginia Institute for Psychiatric and Behavioral Genetics, Virginia Commonwealth UniversityRichmond, VA, USA; ^2^Department of Psychiatry, Virginia Commonwealth UniversityRichmond, VA, USA; ^3^Department of Human and Molecular Genetics, Virginia Commonwealth UniversityRichmond, VA, USA; ^4^Center for Clinical and Translational Research, Virginia Commonwealth UniversityRichmond, VA, USA; ^5^Department of Psychology, Virginia Commonwealth UniversityRichmond, VA, USA; ^6^Department of African-American Studies, Virginia Commonwealth UniversityRichmond, VA, USA; ^7^Faculty of Business, Karabuk UniversityKarabuk, Turkey; ^8^College Behavioral and Emotional Health Institute, Virginia Commonwealth UniversityRichmond, VA, USA

**Keywords:** alcohol problems, alcohol consumption, GWAS, heritability, genetic ancestry, genome-wide polygenic score

## Abstract

**Background:** Genetic factors impact alcohol use behaviors and these factors may become increasingly evident during emerging adulthood. Examination of the effects of individual variants as well as aggregate genetic variation can clarify mechanisms underlying risk.

**Methods:** We conducted genome-wide association studies (GWAS) in an ethnically diverse sample of college students for three quantitative outcomes including typical monthly alcohol consumption, alcohol problems, and maximum number of drinks in 24 h. Heritability based on common genetic variants (*h*^2^_SNP_) was assessed. We also evaluated whether risk variants in aggregate were associated with alcohol use outcomes in an independent sample of young adults.

**Results:** Two genome-wide significant markers were observed: rs11201929 in *GRID1* for maximum drinks in 24 h, with supportive evidence across all ancestry groups; and rs73317305 in *SAMD12* (alcohol problems), tested only in the African ancestry group. The *h*^2^_SNP_ estimate was 0.19 (SE = 0.11) for consumption, and was non-significant for other outcomes. Genome-wide polygenic scores were significantly associated with alcohol outcomes in an independent sample.

**Conclusions:** These results robustly identify genetic risk for alcohol use outcomes at the variant level and in aggregate. We confirm prior evidence that genetic variation in *GRID1* impacts alcohol use, and identify novel loci of interest for multiple alcohol outcomes in emerging adults. These findings indicate that genetic variation influencing normative and problematic alcohol use is, to some extent, convergent across ancestry groups. Studying college populations represents a promising avenue by which to obtain large, diverse samples for gene identification.

## Introduction

Alcohol use phenotypes are genetically influenced, with heritability for alcohol use disorder typically estimated at around 50% in twin and adoption studies (Heath et al., 1997; McGue, 1999; Prescott and Kendler, 1999; Verhulst et al., 2015). Other alcohol-related phenotypes, such as initiation of use, typical consumption, maximum number of drinks consumed in a day, or initial response to alcohol, are also modestly to moderately heritable (Fowler et al., 2007; Poelen et al., 2009; Agrawal et al., 2011; Kalu et al., 2012). As these measures constitute steps along the trajectory to alcohol problems among some individuals, elucidating their genetic etiology is important.

Many genome-wide association studies (GWAS) have been conducted for diagnostic alcohol outcomes (e.g., alcohol abuse/dependence or symptom count; Treutlein et al., 2009a; Bierut et al., 2010; Edenberg et al., 2010; Kendler et al., 2011b; Wang et al., 2013; Gelernter et al., 2014). Overall, these investigations have not enjoyed the same success in large-scale gene-identification efforts as, for example, schizophrenia (Schizophrenia Working Group of the Psychiatric Genomics Consortium, 2014). Quantitative phenotypes are more statistically powerful than binary outcomes in general population samples and have also been the subject of recent genome-wide studies (e.g., Heath et al., 2011; Schumann et al., 2011; Pan et al., 2013; Kos et al., 2014; Edwards et al., 2015). In some cases, associations surpassing stringent genome-wide significance criteria have been observed (Schumann et al., 2011; Pan et al., 2013), though robust replication has not been reported.

Although, robust and replicated variant level associations have yet to be discovered for alcohol related measures, most GWAS have supported the hypothesis that these phenotypes are classic quantitative genetic traits, influenced by many variants (hundreds to thousands) of individual small effect. Methods that examine the aggregate effects of common variants represent an important complement to analyses based on single markers (e.g., primary GWAS results) and support the results from twin and family studies. Heritabilities based on common markers using unrelated individuals have been reported including an alcohol problems score in Dutch adults (0.33; Mbarek et al., 2015), and maximum drinks in 24 h and alcohol use disorder (AUD) (0.32 and 0.34, respectively; Kos et al., 2014) in a modestly sized sample of Mexican-American adults. However, in a UK sample of emerging adults, a heritability estimate of 0.05 for alcohol problems was non-significant (Edwards et al., 2015).

Most prior GWAS have focused on alcohol outcomes in mature adults, and less is known about the transition period between adolescence and young adulthood. Alcohol use increases across adolescence, with consumption, risky drinking, and alcohol use disorders all peaking in young adulthood (SAMHSA, 2014). Longitudinal studies indicate that heritability estimates for alcohol use and problems also increase across adolescence and generally reach the levels observed in adults during emerging adulthood (Rose et al., 2001; Palmer et al., 2013; Samek et al., 2013), though there is some suggestion from cross-sectional and/or retrospective studies that heritability may continue to increase slightly across adulthood (Bergen et al., 2007; Kendler et al., 2007; Hansell et al., 2008). During emerging adulthood there is also a shift in the nature of genetic influences on substance use outcomes, as it is during this time frame that genetic factors become more substance-specific (Vrieze et al., 2012) and less related to overall externalizing behavior (Kendler et al., 2011a; Edwards and Kendler, 2013; Meyers et al., 2014). Thus, emerging adulthood is a critical time frame for clarifying alcohol use etiology.

Here, we examine problematic and normative alcohol use in a population-based sample of US emerging adults. The Spit for Science (S4S) sample (Dick et al., 2014) was recruited at a large, urban university as part of a study on alcohol use and other health-related behaviors. In addition to emerging adulthood being an important period for clarifying the etiology of alcohol use outcomes, there is evidence that college students drink more than their non-college attending peers (Quinn and Fromme, 2011), and that the college environment actually enhances the degree to which genetic influences are important for alcohol consumption (Timberlake et al., 2007). Accordingly, this population presents an opportunity to readily obtain large samples in order to assess genetic influences on alcohol outcomes. The Spit for Science sample's ethnic diversity further enables us to assess whether genetic effects are consistent across ancestry groups.

Three cross-sectional alcohol outcomes were assessed: typical consumption, maximum drinks in 24 h, and a quantitative measure of alcohol problems, which have been demonstrated to be phenotypically and genetically correlated (Kendler et al., 2010; Dick et al., 2011). A series of analyses investigate the effects of individual variants as well as aggregate measures of genetic risk. These effects are tested for replication in an independent sample.

## Methods

### Sample

Spit for Science is an ongoing longitudinal study of college students enrolled in a large, urban university in the Mid-Atlantic, as previously described (Dick et al., 2014). Briefly, incoming students age 18 or older were eligible to complete phenotypic assessments, which covered a wide range of topics but focused on alcohol use. Study data were collected and managed using REDCap electronic data capture tools (Harris et al., 2009) hosted at Virginia Commonwealth University. Follow-up assessments were completed in subsequent spring semesters. Individuals who did not participate in the first wave of data collection (including those who turned 18 after the end of the first wave of data collection) had the opportunity to join the study the following spring; those who participated during their first year were eligible to complete follow-up assessments each spring. Participants who completed the phenotypic assessments were eligible to provide a DNA sample. The current study includes three cohorts, which matriculated in Fall 2011 (*N* = 2,714), 2012 (*N* = 2,486), and 2013 (*N* = 2,403), for a total *N* = 7,603. Of these, 98% provided a DNA sample. As the current analyses are based on the data capture after the Spring 2014 survey, data were available for 2–4 waves, depending on cohort. At wave 1 (61% female), the average (*SD*) age was 18.59 (0.61); at wave 2 (65% female), 18.99 (0.44); at wave 3 (66% female), 19.94 (0.60); and at wave 4 (66% female), 20.93 (0.69).

### Alcohol use variables

For the current study, three alcohol-related variables were derived including a measure of past 30-day alcohol consumption in grams of ethanol (Consumption), the lifetime maximum number of drinks consumed in a 24-h period (Maxdrinks), and an alcohol problems sum score (Symptoms). Abstainers were not administered the alcohol-related items and therefore were not included in any of the current phenotypic and genetic analyses. Participants were given the option of skipping questions therefore number of participants varies across constructs. When a participant had assessments from different waves, the highest score was used.

#### Typical consumption (consumption)

Participants were asked to report on their drinking in the past month with items regarding how frequently they drank and how many drinks they typically consumed on a drinking day. These items were combined to create a single measure of grams of ethanol consumed in the last month, using a method previously described (Dawson, 2000) and reported for the current sample by Salvatore et al. (2016). Briefly, the frequency and quantity variables were multiplied, and that product was multiplied by 14 (corresponding to the number of grams of ethanol in a standard alcoholic drink). Responses were windsorized with a maximum value of 2,000 g to account for unlikely responses. Values were natural log transformed (after adding 1 to each value). Phenotypic data were available for 7,374 participants.

#### Alcohol problems sum score (problems)

In waves 1–4, participants who reported having ever consumed alcohol were asked items related to DSM-5 (American Psychiatric Association, 2013) alcohol use disorder criteria (e.g., “Have you ever started drinking and become drunk when you didn't want to?”), with some criteria assessed using multiple items. For all but 2 items, response options were “never,” “1–2 times,” or “3 or more times,” which were scored 1, 2, and 3, respectively. Items addressing craving and tolerance had response options of “no” and “yes,” coded 0 and 1, respectively. Participants missing more than half their data within a wave were coded as missing for that wave. For each wave, sum scores were created and pro-rated to account for the missingness (when at least half the data was present) and data structure. For the first survey of wave 1, only the seven DSM-IV alcohol dependence items were assessed. None of the four alcohol abuse items were measured. Subsequent surveys of wave 1 and all surveys for wave 2–4 included 11 DSM-V items which were modified to make them appropriate for the participants in accordance with IRB guidelines that the language be written at a 10th grade reading level. The highest pro-rated score across all available waves was selected and natural log-transformed (after adding 1), with data available for 6,082 participants.

#### Maximum drinks in 24 h (maxdrinks)

Participants were asked in waves 1–4 to report the highest number of drinks they had ever consumed in a 24-h period, with response options ranging from 1 to 24, plus “more than 24.” They could also opt not to answer or select “I don't know,” both of which were recoded as missing for the current analyses. Data were available for 6,125 participants. We selected the highest reported Maxdrinks for each individual, as well as the participant's age at that report for inclusion as a covariate in genetic analyses.

### Genotyping, pre-imputation QC, and imputation

There were 6534 samples passing DNA and initial genotyping QC. Genotyping was performed at Rutgers University Cell and DNA Repository (RUCDR) using the Affymetrix BioBank array (653 k variants) which contains both common GWAS framework variants (296 k) for imputation and functional variants (357 k) including rare high impact exome variants (272 k), indels (18 k), eQTLs (16 k), and miscellaneous (51 k). QC excluded Off Target Variants found by SNPolisher, single nucleotide polymorphisms (SNPs) missing >5% of genotypes, samples missing >2% of genotypes, and SNPs missing >2% of genotypes after sample filtering, similar to the Psychiatric Genomics Consortium (PGC; Schizophrenia Working Group of the Psychiatric Genomics Consortium, 2014). This pre-imputation QC removed 209 samples, leaving 6,325 samples and 560,138 variants for imputation. Imputation was conducted using SHAPEIT2 (Delaneau et al., 2013)/IMPUTE2 (Howie et al., 2009) and the 1000 genomes phase 3 reference panel (*n* = 2,504) (1000 Genomes Project Consortium et al., 2015; Sudmant et al., 2015).

### Ancestry principal components (PC)

1000 Genomes Project (1KGP) phase 3 variants (2,504 samples, 26 populations) found in common with the post QC filtered S4S genotypes were merged together. Regions with high LD were excluded (Price et al., 2006, 2008) and the common set of variants was then pruned (*r*^2^ < 0.1) using PLINK 1.9 (Purcell et al., 2007; Chang et al., 2015) (–indep-pairwise 1,500 150 0.1) to yield 109,259 semi-independent variants for ancestry analyses. EIGENSOFT/SmartPCA (Patterson et al., 2006; Price et al., 2006) was used to perform PCA using only the 1KGP phase 3 reference panel to determine SNP weights for each eigenvector. This solution was then projected onto the S4S data to generate 10 principal components (PCs).

### Genetic based population assignment

S4S is ethnically diverse with self-identified census race/ethnicity as follows: American Indian/Alaska Native (*N* = 35); Asian (*N* = 1223); Black/African American (*N* = 1464); Hispanic/Latino (*N* = 450); More than one race (*N* = 467); Native Hawaiian/Other Pacific Islander (*N* = 50); Unknown (*N* = 30); and White (*N* = 3763). Participants could also elect not to answer (*N* = 108). As noted previously, the sample of participants corresponds closely to the overall demographics of the university student population (Dick et al., 2014).

For genetic analyses, S4S subjects were empirically assigned to 1KGP based ancestry super-populations. Briefly, using all 10 ancestry PCs, the Mahalanobis distance (Mahalanobis, 1936) between each S4S sample and each 1KGP population (*N* = 26) without reference population outliers (>4 *SD* from population median, *N* = 61) was calculated. Each subject was then assigned to the 1KGP population with the minimum Mahalanobis distance and then collapsed into their respective super-population assignment. This empirically based ancestry has several advantages to self-identified race/ethnicity including reducing variance of the within group PCs and being able to include “Unknown,” “More than one race,” and small groups in the analysis without an increase in genomic inflation. There were five final ancestry groups: African descent (AFR), American descent (AMR), East Asian descent (EAS), European descent (EUR), and South Asian descent (SAS).

### Within group quality control

Due to the diverse nature of S4S, filtering by Hardy-Weinberg Equilibrium (HWE), minor allele frequency (MAF), and relatedness were performed within empirically assigned super-populations. Genome-wide IBD ($\hat{\Pi }$) was calculated using PLINK 1.9. For each sample, the mean cross-sample $\hat{\Pi }$ was calculated to find samples showing excessive relatedness, which is where a sample appears to be a cryptic relative to many other samples but those samples do not appear related to one another. One hundred and ninety four samples were excluded (>2.5 standard deviations above the mean) as outliers for average relatedness with all other samples. Clusters of probable relatives were defined using $\hat{\Pi }$ > 0.1, Z0 >= 0.825, and Z1 < 0.175. The inclusion of Z0/Z1 is important since $\hat{\Pi }$ > 0.1 can be due to artifacts where Z2 > 0 which is extremely unlikely for cryptic relatives. Then the best performing sample for each relative cluster was retained which resulted in an additional 180 samples being excluded from the GWAS sample.

The choice of ancestry PC covariates to include in each super-population GWAS was determined by stepwise linear regression for the specific phenotype being analyzed. Non-ancestry covariates (sex and age) were forced to be kept in the model while ancestry covariates were kept if they were retained in the best fitting model as measured by AIC. This approach, of only keeping PCs significant to a specific ancestry and phenotype, increases parsimony and minimizes risk of over-fitting. We have shown that there is no evidence of genomic inflation when this parsimonious approach is taken.

SNPTEST (Marchini et al., 2007) v2.5.2 was used to conduct association analyses under an additive model only including markers with a minimum MAF of 0.005 and INFO of 0.5. Post GWAS filtering was performed using ancestry specific HWE (*p* > 10^−6^) and sample size based MAFs. Instead of using a fixed MAF threshold for each group, the minimum observed minor allele count (MAC) was used. Previous research has shown a MAC of ~40 is robust for most association analyses performed in GWAS (Bigdeli et al., 2014). Post filtered GWAS results were meta-analyzed using METAL (Marchini et al., 2007; Willer et al., 2010) which implements a fixed effect model and inverse variance weighting based on sample size. Markers available for fewer than 1,000 individuals after meta-analysis were excluded. Estimation of genomic inflation (λ, λ_1,000_) for within super-population GWAS and meta-analyses was performed in R (R Core Team, 2016). False Discovery Rate (FDR) analysis was performed using the “*q*-value” package (https://github.com/jdstorey/qvalue) using Bioconductor 3.2 (Huber et al., 2015). To define genomic bins for follow-up, we started with all markers with a *q* < 0.5. Markers were initially collapsed into bins if they were within 10 kb. *Post-hoc* inspection showed several adjacent bins <75 kb apart which were then collapsed into the reported bins.

### GCTA

Genome-wide Complex Trait Analysis was used (Yang et al., 2011) (GCTA) to estimate the proportion of phenotypic variance attributable to observed (non-imputed) genetic variants [V(G)/Vp, or *h*^2^_SNP_]. Genetic relationship matrices (GRMs) were derived for each ancestry group. Within ancestry group, only unrelated individuals were included in the GRM as in the GWAS, resulting in the following sample sizes: AFR: *N* = 1339; AMR: *N* = 582; EAS: *N* = 557; EUR: *N* = 3018; SAS: *N* = 455. An ancestry group-specific MAF cutoff of 0.01 was applied. The same ancestry PCs used in the GWAS analyses were included in the heritability analyses.

### Replication

#### Sample

We used the Avon Longitudinal Study of Parents and Children (ALSPAC; Boyd et al., 2013; Fraser et al., 2013) to test for replication of individual variants as well as at the aggregate level. ALSPAC participants were born in 1991–1992 and are predominantly of European descent (>96%). The total ALSPAC sample included 15,247 pregnancies from women residing in Avon, UK with expected due dates between April 1991 and December 1992, resulting in 15,458 fetuses. Of this total sample, 14,775 were live births and 14,701 were alive at 1 year of age. The study website contains details of all the data that are available through a fully searchable data dictionary (http://www.bris.ac.uk/alspac/researchers/data-access/data-dictionary/). Ethical approval for the study was obtained from the ALSPAC Ethics and Law Committee, Bristol University and Virginia Commonwealth University. Genetic data are available for *N* = 8,237 individuals who meet quality control filters.

#### Phenotypes

ALSPAC participants were administered a self-report questionnaire that included alcohol use items at approximately age 20.75. These items included questions about frequency of alcohol use and average number of drinks per drinking day, from which the Consumption variable is derived; DSM-IV and AUDIT (Babor et al., 2001) items, from which a problems sum score is derived; and maximum number of drinks in 24 h. Thus, they corresponded quite closely to those administered to S4S participants. Sample size varied across outcomes (Consumption *N* = 3,150, Problems *N* = 2,906, and Maxdrinks *N* = 2,670) as a function of attrition and alcohol use initiation.

#### Genotyping

The ALSPAC sample was genotyped on the Illumina HumanHap550 quad SNP genotyping platform by the Wellcome Trust Sanger Institute (Cambridge, UK) and Laboratory Corporation of America (Burlington, NC, US). Samples were subjected to quality control filters as previously described (Edwards et al., 2015). Data were imputed using a phased version of the 1000 Genomes reference panel (Phase 1, Version 3), using Impute V2.2.2 and all reference haplotypes to maximize imputation quality.

#### Individual variant replication

For markers with *q* < 0.5 in the three meta-analysis results, we extracted summary statistics from GWAS results of the corresponding phenotype in ALSPAC. Due to differences in allele frequencies across the discovery and replication samples, summary statistics were not available for all markers.

#### Genome-wide polygenic scores (GPS)

*P*-value threshold based polygenic scores (GPS) were calculated in ALSPAC using the S4S GWAS results. Prior to GPS derivation, we identified markers present in both the S4S and ALSPAC genetic data with MAF > 0.01 and INFO > 0.5. We pruned this list using the clumping function in PLINK 1.9 (Chang et al., 2015) to identify independent loci. Clumping was conducted based on the European subsample of the 1000 Genomes reference panel, in two stages: the first used an *r*^2^ threshold of 0.5 and a range of 250 kb; the second used an *r*^2^ threshold of 0.1 and a range of 5 mb. Markers selected were used to derive GPS for ALSPAC participants, employing a series of *p*-value thresholds as is customary. We derived GPS based on the Z-scores (and associated *p*-values) from the METAL meta-analyses as well as based on EUR-specific SNP regression coefficients (and associated *p*-values), given the possibility that the latter might be more predictive of outcome in the European ALSPAC sample. In R (v3.2.3), we tested whether GPS were associated with alcohol outcomes (Consumption, Problems, or Maxdrinks), controlling for sex, using linear regression. Our analytic goal was to test for association rather than to try to predict outcome, given the sample sizes and expected genetic effect sizes (Dudbridge, 2013).

## Results

### Descriptive statistics

Mean values for each variable are reported in Table 1. Variables were moderately inter-correlated (*r* = 0.42–0.58). The sample consists of more women than men (60.8% female), though this distribution is only slightly different than that of the student population. Women reported lower levels of drinking for all outcomes (*p* < 0.01). Sex was included as a covariate in genetic analyses.

**Table 1:** **Descriptive statistics for untransformed alcohol outcome variables**.

**Alcohol phenotype**	**Mean (*SD*)**	**Range**	**Correlations**		
			**Consumption**	**Problems**	**Maxdrinks**
Consumption^a^	262.34 (437.26)	0–2,000	1		
Problems^b^	20.59 (5.32)	16–48	0.47	1	
Maxdrinks	9.81 (6.07)	1–30	0.58	0.42	1

a *Values presented are for scores after imposing a cutoff at 2,000 g, prior to log transformation*.

b *Values presented are prior to log transformation*.

### GCTA/heritability estimates

We used GCTA to calculate *h*^2^_SNP_ for each of the three phenotypes, separately by ancestry group. Results are provided in Table 2. We next meta-analyzed these results across ancestries, as several groups (AMR, EAS, and SAS) had limited sample sizes. Meta-analytic results indicated that Consumption is modestly heritable, while estimates for Problems and Maxdrinks were not significantly different from zero at this age.

**Table 2:** **SNP-based heritability estimates**.

**Alcohol outcome**	**Ancestry group**	***N***	***h*****^2^_SNP_ (SE)**	***p*****-value**	**Meta-analysis *h*^2^_SNP_ (SE)**
Consumption	AFR	1,291	<0.01 (0.27)	0.50	0.19 (0.11)
	AMR	557	0.35 (0.43)	0.24	
	EAS	534	0.46 (0.52)	0.13	
	EUR	2,899	0.22 (0.13)	0.04	
	SAS	446	<0.01 (0.67)	0.50	
Problems	AFR	1,053	<0.01 (0.15)	0.50	0.02 (0.10)
	AMR	466	0.17 (0.59)	0.40	
	EAS	409	1.00 (0.86)	0.10	
	EUR	2,561	<0.01 (0.15)	0.50	
	SAS	292	<0.01 (0.65)	0.50	
Maxdrinks	AFR	1,076	<0.01 (0.29)	0.50	0.01 (0.12)
	AMR	474	<0.01 (0.54)	0.50	
	EAS	414	<0.01 (0.58)	0.50	
	EUR	2,566	0.02 (0.14)	0.45	
	SAS	283	<0.01 (0.99)	0.50	

*AFR, African; AMR, Ad Mixed American; EAS, East Asian; EUR, European; SAS, South Asian*.

### Primary GWAS results

After applying filtering and meta-analysis, results were available for 16,511,702, 15,625,945, and 15,724,050 markers for Consumption, Problems, and Maxdrinks, respectively. For each phenotype, results for 33–35% of the markers were only testable (MAC > 40) in the AFR ancestry group due to frequency. The meta-analyses showed no evidence of genomic inflation with λ_1,000_s of 1.0001 (Problems), 0.9997 (Consumption), and 0.9994 (Maxdrinks). Across the three phenotypes, FDR analysis showed 187 markers with *q* < 0.50. All markers with FDR *q* < 0.5 are listed in Supplementary Table 1, with summary statistics and information on nearby genes. These 187 markers map to 53 genomic bins (Supplementary Table 1). Seven bins contained at least one genome-wide significant (GWS; *p* < 5 × 10^−8^) marker. However, in all but one of these bins the signal was limited to the AFR group. Using stricter genome-wide significance for samples of African ancestry (*p* < 1 × 10^−8^), only one AFR specific bin remained GWS.

**Figure 1:**
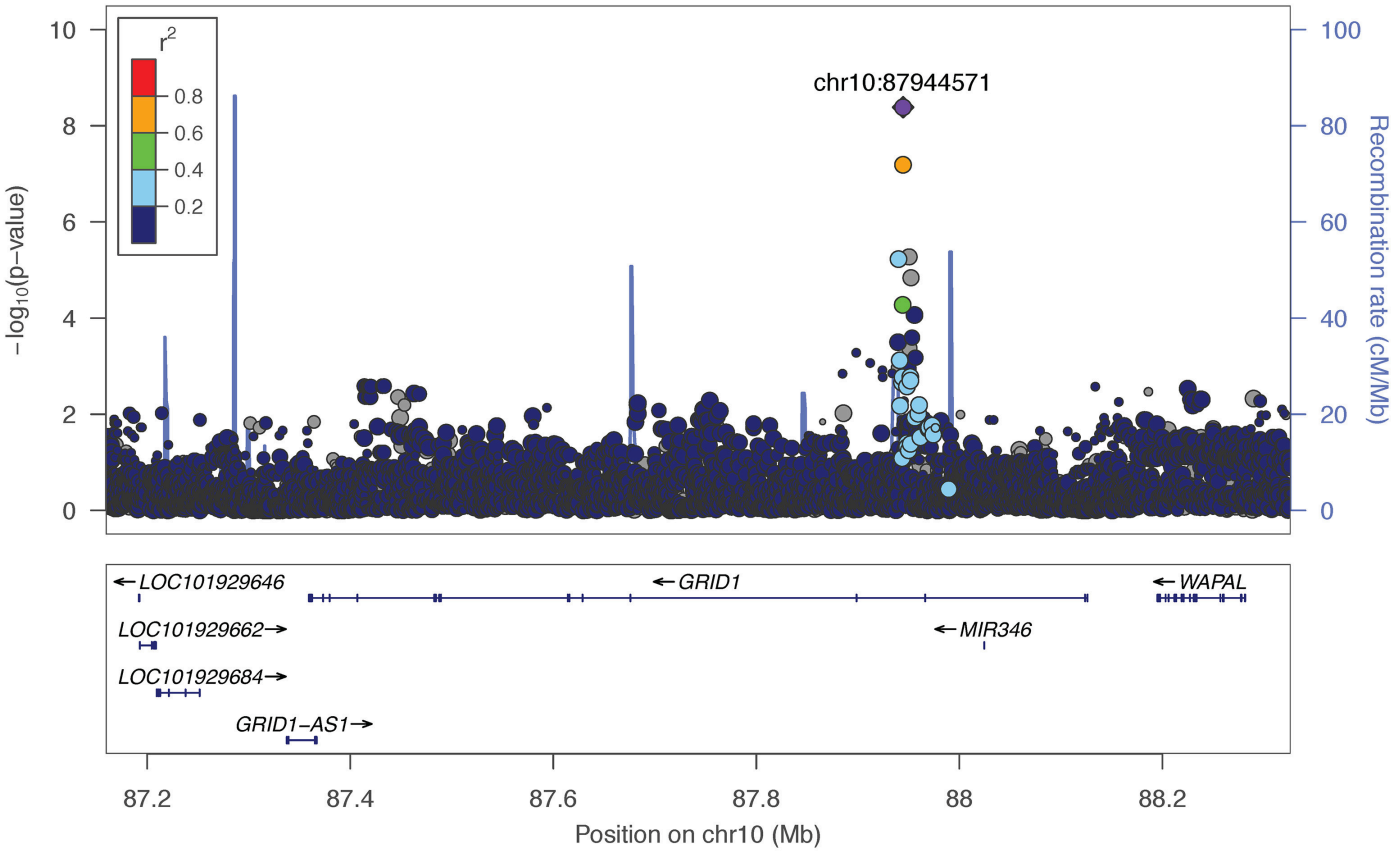
**Regional association plot for *GRID1* and 200 kb flanking regions, implemented using LocusZoom (Pruim et al., 2010)**. The most significant marker is in purple (rs11201929, *p* = 4.11e-09, *q* = 0.06 for Maxdrinks). Linkage disequilibrium information is based on the 1000 Genomes AFR super-population. The size of the points representing plotted SNPs corresponds to the meta-analysis sample size.

**Figure 2:**
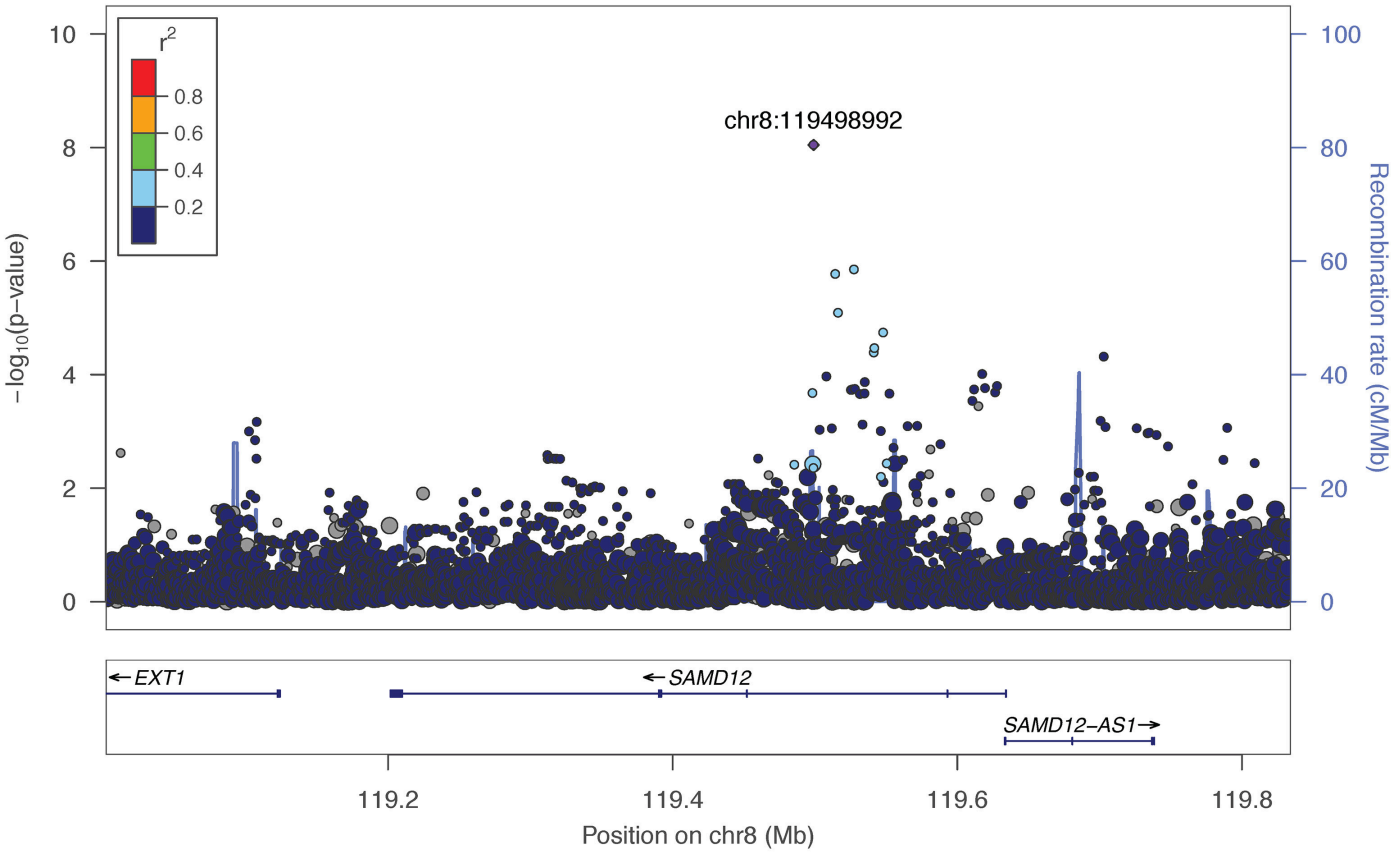
**Regional association plot for *SAMD12* and 200 kb flanking regions**. The most significant marker is in purple (rs73317305, *p* = 9.02 × 10^−9^, *q* = 0.11 for Problems). Linkage disequilibrium information is based on the 1000 Genomes AFR super-population, as the minor allele was rare in other subgroups. The size of the points representing plotted SNPs corresponds to the meta-analysis sample size.

The two markers robustly GWS are rs11201929 for Maxdrinks (*p* = 4.11 × 10^−9^, *q* = 0.06) and rs73317305 for Problems (*p* = 9.02 × 10^−9^, *q* = 0.11). rs11201929 is in the third intron of *glutamate ionotropic receptor delta type 1* (*GRID1*) and was of sufficient frequency and quality to be tested in all five ancestry groups. Figure 1 depicts the regional association plot for *GRID1*. The direction of effect was consistent across ancestries, though the strength of the association varied (rs11201929 *p*-values by group: 5 × 10^−7^ AFR, 0.046 AMR, 0.07 EAS, 0.0067 EUR, 0.13 SAS). The other GWS marker, rs73317305 (Problems), is in the second intron of *sterile alpha motif domain containing 12* (*SAMD12*) on chromosome 8 and is rare in non-African populations (MAFs: AFR 0.0898, AMR 0.0175, EAS 0, EUR 0.0021, SAS 0.0008; Figure 2).

For Consumption, the most strongly associated marker was rs76541530 (*p* = 3.39 × 10^−8^, *q* = 0.10), which does not meet our stringent genome-wide significance criterion for variants informative only in the AFR group (*p* < 1 × 10^−8^). This marker, along with 12 others that map to the same genomic region on chromosome 11, does not map to any known gene.

### Replication in ALSPAC

For replication, we considered samples similar to S4S with respect to age and/or alcohol phenotypes in order to minimize lack of replication due to differences in ascertainment and phenotypes. ALSPAC met these criteria due to being a sample similar in age and the derived variables being nearly identical. Replication was attempted for (1) individual variants with FDR *q* < 0.50 and (2) *p*-value threshold based polygene scores. Importantly, only results based on equivalent phenotypes were examined (e.g., markers associated with Problems in S4S were not examined for their association with Maxdrinks or Consumption). While several variants with *q* < 0.50 exhibited nominal associations (*p* < 0.05) in ALSPAC, we did not observe robust variant level replication after correction for multiple testing for any phenotype. Results are presented in Supplementary Table 2. In contrast to specific SNP results, each GPS showed some evidence for replication, with support varying across outcomes. Consumption and Problems both showed nominal significance (*p* < 0.05) for a wide (p_threshold_ 0.01 to 0.5) and narrow range (p_threshold_ 0.01) of thresholds, respectively. The Maxdrinks PRS was robustly associated (*p* < 0.005) across a wide range of thresholds (p_threshold_ 0.05–0.5) Further details are provided in Table 3. Variance accounted for by the scores was low (<1%) for Consumption and Problems; for Maxdrinks scores accounted for >6% of the variance in some cases. Scores derived from the EUR-specific GWAS were similar though less pronounced (Supplementary Table 3).

**Table 3:** **Associations between GPS derived from S4S meta-analysis results and ALSPAC alcohol outcomes**.

**Threshold**	**Consumption**			**Problems**			**Maxdrinks**		
	**Beta**	***p*****-value**	***r*****^2^**	**Beta**	***p*****-value**	***r*****^2^**	**Beta**	***p*****-value**	***r*****^2^**
*p* < 0.0001	0.0005	0.5844	0.0001	<0.0001	0.8548	<0.0001	0.0099	0.1127	0.0055
*p* < 0.001	<−0.0001	0.9379	<0.0001	<0.0001	0.6083	0.0001	0.0023	0.3009	<0.0001
*p* < 0.01	0.0002	0.0715	0.0013	0.0001	0.0088	0.0029	0.0011	0.2031	0.0037
*p* < 0.05	0.0002	0.0038	0.0037	<0.0001	0.0107	0.0022	0.0013	0.0050	0.0255
*p* < 0.10	0.0001	0.0093	0.0036	<0.0001	0.0960	0.0007	0.0011	0.0025	0.0675
*p* < 0.20	0.0001	0.0173	0.0028	<0.0001	0.1772	0.0007	0.0011	0.0008	0.0620
*p* < 0.30	0.0001	0.0148	0.0028	<0.0001	0.1070	0.0009	0.0010	0.0008	0.0649
*p* < 0.40	0.0001	0.0126	0.0029	<0.0001	0.1029	0.0011	0.0010	0.0007	0.0621
*p* < 0.50	0.0001	0.0114	0.0030	<0.0001	0.0985	0.0011	0.0010	0.0010	0.0533

*Nagelkerke's r^2^ is provided as a measure of variance accounted for by the polygenic score*.

## Discussion

Using a population-based study of emerging adults at a diverse mid-Atlantic university, the Spit for Science sample, we present evidence of replicable aggregate genetic influences on three alcohol-related phenotypes: typical monthly consumption (Consumption), maximum drinks in 24 h (MaxDrinks), and an alcohol problems sum score (Problems). We find further support that Consumption is heritable (*h*^2^_SNP_ 0.19, SE = 0.11). At the marker level, variation in a previously implicated (see below) gene, *GRID1*, surpasses stringent genome-wide significance criteria for association with MaxDrinks. Furthermore, polygenic scores derived from S4S for Consumption and Maxdrinks, and Problems to a lesser extent, are significantly associated with the equivalent outcomes in an independent and comparably-aged sample. These results provide empirical support for the influence of aggregate molecular variation on multiple alcohol outcomes.

We observed associations between alcohol phenotypes and markers that localize to within or near genes of biological interest. Most notable is the association between Maxdrinks and the *glutamate ionotropic receptor delta type subunit 1* gene (*GRID1*), which is involved in synaptic plasticity. *GRID1* has been implicated in prior genetic studies of alcohol use outcomes: Chen et al. (2015) found that SNPs nominally associated with alcohol cue-elicited brain activation were enriched for markers mapping to genes, including *GRID1*, that are involved in synaptic long term depression. This gene was further implicated in comorbid alcohol dependence and depressive syndrome (Edwards et al., 2012), and in a study of alcohol problems in a population-based sample (Edwards et al., 2015). Glutamatergic receptor subunit mRNA, including GRID1, has been shown to be altered in the caudate within an alcoholic sample relative to controls (Bhandage et al., 2014). More generally, *GRID1* has been associated at varying levels of significance with brain structure (Nenadic et al., 2012) and schizophrenia (Fallin et al., 2005; Treutlein et al., 2009b; Nenadic et al., 2012). Besides *GRID1*, top markers mapped to within or near *NPAS3*, which potentially regulates genes involved in neurogenesis and has been previously associated with psychiatric disorders (Pickard et al., 2006; Huang et al., 2010; Nurnberger et al., 2014); and *SV2B*, a synaptic vesicle protein-encoding gene implicated in cognitive processes in model systems (Detrait et al., 2014; Olson et al., 2015).

There was not support for specific (*q* < 0.5) variants in another sample assessed for comparable alcohol use phenotypes. Such failures to replicate may be due to population-specific effects (genetic and/or environmental) or false positive results. Many markers with *q* < 0.5 in the current analyses were assayed only in the AFR ancestry group due to low MAC in other groups. These results underscore the need for additional genetic analyses to be conducted in samples of non-European ancestry, not only to facilitate replication, but also to clarify potential differences in genetic risk factors across ancestries (Dick et al., 2017).

We find that GPS derived from markers associated with ethanol Consumption and MaxDrinks at modest *p*-value thresholds (*p* < 0.05 and above) predict those same outcomes in the ALSPAC sample. These results are consistent with the highly polygenic nature of alcohol phenotypes. Although GPS derived from relatively few markers (several hundred to ~25,000 for up to *p* < 0.01) were not predictive of outcome, those that are more inclusive do capture meaningful genetic liability. Furthermore, this interpretation is consistent with the null results of our replication attempts for markers with *q* < 0.50. Thus, it is likely that many of the variants implicated at marginal thresholds are incrementally contributing to risk, though to a degree too small to be detected in isolation. We observe similar trends when GPS are derived from the European ancestry group only, though results are less robust (Supplementary Table 3). We likely benefitted from the improved statistical power of the meta-analysis. These findings also provide support for the hypothesis that genetic variants impacting alcohol outcomes are largely similar across different ethnicities, though given variation with respect to allele presence/frequencies there are also likely to be ethnicity-specific genetic factors.

Our estimate of the heritability of consumption was far lower than estimates (Manolio et al., 2009; Zuk et al., 2012) obtained previously for consumption in young adults in previous studies (Rose et al., 2001; Palmer et al., 2013). Current methods used to calculate *h*^2^_SNP_ typically result in estimates that are lower than those obtained using traditional biometric modeling. For example, a recent study of a Dutch sample assessed using the AUDIT reported a twin-based *h*^2^ estimate of 0.6, with a corresponding SNP-based estimate of *h*^2^_SNP_ 0.33 (Mbarek et al., 2015). Such findings (“missing heritability”) have been discussed extensively (Manolio et al., 2009; Lee et al., 2011; Zuk et al., 2012; Brookfield, 2013; Koch, 2014), and may be due to rare variants, poor tagging of common variants, overestimation of heritability in twin studies, epistatic interactions, epigenetic factors, or other genomic phenomena. Accordingly, the low and non-significant *h*^2^_SNP_ estimates for Problems and Maxdrinks may reflect the presence of other genetic factors, insufficient statistical power, or both (Supplementary Table 4). There are other potential explanations specific to the study and outcomes. First, recall bias is potentially an issue for Maxdrinks since ~50% of drinkers in our sample report having blacked out when drinking. There is also an overall elevation in misuse and associated problems during this age range which may reflect more environmental factors and mask genetic variation. Additionally, reports of alcohol misuse and associated consequences may reflect a certain degree of bravado at this age, when some youth may have less developmental perspective on alcohol problems. It is likely inappropriate to interpret these results as an indication that genetic factors do not impact alcohol Problems and Maxdrinks in this population, particularly given the positive associations between S4S-derived GPS and alcohol outcomes in an independent sample. Indeed, non-significant *h*^2^_SNP_ estimates can mask meaningful genetic variation that simply does not account for a substantial component of phenotypic variance. In the current study, the rs671 variant within *ALDH2*, which is common in East Asian populations and is known to impact risk for AUD (Edenberg, 2007), was strongly associated with all three outcomes within the EAS-specific results (p_Consumption_ 0.013, p_MaxDrinks_ 0.00014, p_Symptoms_ 0.0039). Nonetheless, this did not translate into significant *h*^2^_SNP_ estimates within that ancestry group.

### Limitations

The results presented herein should be considered in light of several limitations. First, the phenotypes examined are based on self-report data, which do not offer the possibility of external verification and are subject to reporting bias. We attempted to mitigate potential issues by imposing cut-offs that allowed for variation in responses while restricting values to a reasonable realm of possibility. Second, several of the ancestry groups (American, East Asian, and South Asian descent) included in these analyses were rather small, resulting in large standard errors around parameter estimates for both individual variant analyses and aggregate tests (e.g., GCTA). While the European and African descent groups were larger and more statistically powerful, substantial sample sizes are necessary to reliably detect low heritability using GCTA, and these results should be interpreted with caution.

Many markers with *q* < 0.50 were evaluated only in the AFR group due to low MAC in other groups. Given that most alcohol-related GWAS have been conducted on samples of European ancestry, these markers were largely unavailable for replication attempts in other samples. Had we calculated *q*-values based only on markers available in the EUR group, or only on those available across all groups, the list of markers for follow-up would have differed, potentially impacting the overall outcome of the SNP-based replication assessments.

GPS parameter estimates are quite modest, indicating that scores would account for little of the total variance in ALSPAC outcomes. In addition, we report uncorrected *p*-values, as the tests are not independent and the appropriate correction approach is not immediately evident.

In spite of the above limitations, these analyses demonstrate that genetic factors are integrally involved in liability to alcohol use in college-aged students, at both individual variant and aggregate levels. Furthermore, the effects of many genetic variants are consistent across ethnicities, suggesting shared biological mechanisms. Critically, replication in an independent UK cohort indicates that the observed genetic effects are generalizable within a similarly aged cohort. These findings validate prior evidence of specific and general genetic effects on alcohol outcomes, and provide nascent support for novel loci that merit additional research.

## Ethics statement

All Spit for Science protocols were approved by the VCU Institutional Review Board (IRB).

## Author contributions

The author contributions to the current research include study design (BW, AE, DD, KK), sample collection (KP, AA, MC, JESavage, ZN), phenotype analyses and QC (JESalvatore, FA, SC), DNA sample processing and QC (CS, BR), genotype QC (BW, VW, JK) statistical and bioinformatic analyses (BW, AE, AW) and manuscript preparation (BW, AE, DD, KK).

### Conflict of interest statement

The authors declare that the research was conducted in the absence of any commercial or financial relationships that could be construed as a potential conflict of interest.
