# Supplementary material for: Molecular Genetic Influences on Normative and Problematic Alcohol Use in a Population-Based Sample of College Students
Source: Front Genet. 2017 Mar 15;8:30. doi: 10.3389/fgene.2017.00030 (PMC5350109; doi:10.3389/fgene.2017.00030)
Supplement: Supplementary file 3 [file Table3.DOCX]

**Supplementary Table 3**. Parameter estimates and p-values for regressions of each ALSPAC alcohol outcome onto genome-wide polygene scores (GPS). Sex was included as a covariate. GPS were based on marker weights from the European-specific genome-wide association analysis in Spit for Science. As is customary, a series of GPS were derived, each based on discovery sample p-values.

|  | **Grams of Ethanol** | | **Problems Score** | | **Maximum Drinks** | |
| --- | --- | --- | --- | --- | --- | --- |
| **Predictor** | **Beta** | **p-value** | **Beta** | **p-value** | **Beta** | **p-value** |
| p<0.0001 | 0.0094 | 0.4279 | -0.0275 | 0.3087 | -0.0833 | 0.6234 |
| p<0.001 | 0.0041 | 0.4780 | -0.0161 | 0.1047 | 0.0150 | 0.8036 |
| p<0.01 | 0.0008 | 0.7530 | 0.0002 | 0.3776 | 0.0051 | 0.8215 |
| p<0.05 | 0.0021 | 0.1430 | 0.0001 | 0.5139 | 0.0183 | 0.1335 |
| p<0.1 | 0.0021 | 0.0606 | <0.0001 | 0.7350 | 0.0163 | 0.0960 |
| p<0.2 | 0.0018 | 0.0445 | <0.0001 | 0.3720 | 0.0143 | 0.0777 |
| p<0.3 | 0.0016 | 0.0555 | <0.0001 | 0.5001 | 0.0130 | 0.0801 |
| p<0.4 | 0.0015 | 0.0543 | <0.0001 | 0.4778 | 0.0125 | 0.0776 |
| p<0.5 | 0.0013 | 0.0760 | <0.0001 | 0.6129 | 0.0134 | 0.0521 |
